# Editorial: Unraveling the physiology of cells and extracellular matrix: Techniques for biochemical and biophysical characterization

**DOI:** 10.3389/fphys.2022.1123223

**Published:** 2023-01-04

**Authors:** Renata Kelly Da Palma, Juan Jose Uriarte, Chelsea M. Magin

**Affiliations:** ^1^ Facultad De Ciencias De la Salud de Manresa, Universitat de Vic-Universitat Central De Catalunya (UVic-UCC), Barcelona, Spain; ^2^ Human Movement and Rehabilitation, Post-Graduate Program Medical School, Evangelic University of Goiás-UniEVANGÉLICA, Anápolis, Brazil; ^3^ Department of Physiology, College of Medicine, University of Kentucky, Lexington, KY, United States; ^4^ Department of Bioengineering, University of Colorado, Denver | Anschutz, Aurora, CO, United States; ^5^ Department of Pediatrics, University of Colorado, Anschutz Medical Campus, Aurora, CO, United States; ^6^ Division of Pulmonary Sciences and Critical Care Medicine, Department of Medicine, University of Colorado, Anschutz Medical Campus, Aurora, CO, United States

**Keywords:** extracellular matrix (ECM), physiology, cell biology, characterization and biological activities, cell-matrix interactions

The dynamic, reciprocal interplay between cells and the extracellular microenvironment manifests in physiological states of homeostasis or disease. The extracellular matrix (ECM) is a supramolecular assembly of structural proteins, glycoproteins, proteoglycans, glycosaminoglycans, and associated molecules, including growth factors, matrix metalloproteinases (MMPs), and MMP-inhibitors (Schuppan et al., 1994; Bosman and Stamenkovic, 2003). Cell-matrix communication occurs mainly through receptor-ligand interactions and often results in signal transduction, which modifies cellular phenotype or function. Conversely, cells are capable of not only using forces to remodel the extracellular microenvironment but can also secrete new proteins to alter its composition (Wakatsuki and Elson, 2002). Complex cell-matrix interactions are enriched by extracellular vesicles carrying ECM- and cytoskeletal-signaling molecules as cargo (Manou et al., 2019). Understanding the molecular mechanisms, biophysical cues, and cell-matrix signaling that drive changes in cellular phenotype and ECM remodeling will enable researchers to better model and understand physiology in homeostasis and disease (Bailey et al., 2018). This topic highlights start-of-the-art techniques for characterizing cell-secreted proteins, how mechanotransduction regulates an invasive phenotype in epithelial cells, and how extracellular vesicles or exosomes influence cell behaviors.

The Nobel Prize in Chemistry, 2022 was awarded to Carolyn Bertozzi, Barry Sharpless, and Morten Meldal for the invention and advancement of click-chemistry, a form of chemistry where reactions occur quickly without undesirable by-products (Chemistry, 2022). Inspired by the pioneering work of Dr. Bertozzi and colleagues, Morey et al. employed a biorthogonal chemical reaction to measure the half-life of cellular proteins in the article titled, “SPAAC Pulse-Chase: A Novel Click Chemistry-Based Method to Determine the Half-Life of Cellular Proteins.” This novel method enables researchers to measure the half-life of newly synthesized proteins using non-radioactive labeling and detection approaches. Briefly, newly synthesized proteins were first labeled (pulsed) with L-azidohomoalanine (AHA), a methionine analog containing a reactive azide group that can selectively and non-destructively incorporate into newly synthesized proteins. Cells were collected at specified times, lysed in the chase media, and proteins of interest were immunoprecipitated. AHA-labeled proteins were next combined with a strained cyclooctyne, such as dibenzocyclooctyne (DBCO) coupled to a fluorescent or biotin probe, in a strain-promoted alkyne-azide cycloaddition (SPACC) reaction. These proteins were then resolved on SDS-PAGE gels. Researchers can use this new technique to determine protein half-life in the SDS-PAGE gels by measuring fluorescence or following transfer to a membrane using the biotin probes. Results showed utility of this new click-chemistry technique in both mammalian and yeast cells. This technique has the potential to extend our understanding of how cells contribute to formation and remodeling of the extracellular microenvironment.

Cells are constantly sensing and responding to mechanical forces and physical properties of the surrounding ECM [19]. The mechanical dynamic forces could influence tumor progression, as suggesting by the paper titled “Mechanotransduction of Strain Regulates an Invasive Phenotype in Newly Transformed Epithelial Cells”. Chagnon-Lessard and colleagues fabricated a microfluid stretch device to apply a dynamic stretch to epithelial cells and quantify resulting cellular invasion. They concluded that mechanical force (stretching) induced a new mechanical configuration of epithelial cells by increasing protrusion formation and reducing apical extrusion, likely regulated by the Rho-ROCK pathway. This study demonstrated the importance of applying mechanical dynamic forces during *in vitro* cancer cell studies.

Extracellular vesicles (EVs) consist of three main subtypes, identified by their size and origin: exosomes (the smallest, released by the fusion of multivesicular bodies with the plasma membrane), microvesicles (secreted by budding from the plasma membrane) and apoptotic bodies (derived from cell rupture) (van Niel et al., 2018). Bone marrow mesenchymal stromal cells (BMSCs) transfer EVs to protect against tissue or organ damage. In the study “Human Bone Marrow Mesenchymal Stromal Cell-Derived Extracellular Vesicles Promote Proliferation of Degenerated Nucleus Pulposus Cells and the Synthesis of Extracellular Matrix Through the SOX4/Wnt/β-Catenin Axis”, the authors demonstrated that hBMSC-EVs promoted degenerated nucleus pulposus cells (DNPCs) proliferation and ECM synthesis by carrying miR-129–5p into DNPCs to target SOX4 and inhibit the activation of the Wnt/β-catenin pathway. This work opens new treatment possibilities for intervertebral disk degeneration.

Exosomes are a special subtype of extracellular vesicles comprised of a phospholipid bilayer surrounding a cargo that may include proteins, small molecules, nucleic acids, and/or other metabolites. These 30–150 nm in diameter messengers are released into the extracellular matrix by cells and have been shown to regulate the behavior of recipient cells. In the manuscript titled, “The regulatory role of exosomes in venous thromboembolism”, Ye et al. review the physiological and biochemical characteristics of exosomes and focus on their potential clinical applications. The role of exosomes as biomarkers and therapeutic vectors in thromboembolism is particularly highlighted in this contribution. The studies reviewed here show that exosome cargoes may influence coagulation, intercellular interactions, and activation of signaling pathways in the thrombosis cascade. The authors conclude with a challenge to researchers in this field to better define mechanisms regulating exosome involvement in pathophysiology.

In conclusion, the collection of articles published in the present Research Topic unravels new aspects of cell physiology as influenced by interactions with the extracellular matrix. These manuscripts characterize cell-secreted ECM proteins, probe how mechanotransduction regulates epithelial cell invasion in cancer, and describe ways in which extracellular vesicles or exosomes influence cell behaviors. Continued research into understanding the biochemical and biophysical cues presented by the ECM, as well as how cell-matrix interactions drive changes in phenotype, will enable researchers to better model and understand human tissues in homeostasis and disease.
